# Supplementary material for: The genome sequence of a green lacewing, Nineta flava (Scopoli, 1763)
Source: Wellcome Open Res. 2024 Aug 14;9:477. [Version 1] doi: 10.12688/wellcomeopenres.22777.1 (PMC11535497; doi:10.12688/wellcomeopenres.22777.1)
Supplement: Peer review history

# Reviewer report: Reviewer response for version 1

**DOI:** 10.21956/wellcomeopenres.25081.r98431

**Published:** 2024-11-04

**Authors:** David Luecke

**Affiliations:** 1 University of California Davis, Davis, USA

This article reports the sequencing and *de novo* assembly of the genome of the green lacewing species *Nineta flava*, and describes the resulting reference assembly. This resource is the first reference genome in the *Nineta* genus and the forth representative of the green lacewings.

 The authors generated PacBio HiFi and Illumina HiC sequencing libraries from the same individual (sex unknown), which provides maximum haplotype phasing ability. However the initial assembly of contigs from HiFi reads with HiFiASM did not incorporate HiC reads and targeted primary and alternate contigs (rather than haplotype 1 and 2), so the phasing capacity is not fully utilized. HiC reads were used for scaffolding of contigs with YaHS, and ultimately generated a highly contiguous chromosome-level scaffolded assembly. Completeness as assayed by the BUSCO endopterygota_odb10 benchmark set is excellent; this is the most appropriate lineage choice for green lacewings. The mitochondrial genome was assembled separately with MitoHiFi, producing a circular sequence of expected size. No attempt was made to recover the identity of sex chromosomes as this requires sex-specific sequence information. All methods are well documented and reference quality is properly tested and reported.

 This is an excellent *de novo* genome reference report. The resulting products (reference and alternate assemblies) are trustworthy inputs for most any downstream application. If long-range linkage information is required a fully phased diploid assembly should be generated from the provided raw HiFi and HiC sequence data. Sex chromosome analysis will require generation of additional sequence data.

Are sufficient details of methods and materials provided to allow replication by others?

Yes

Is the rationale for creating the dataset(s) clearly described?

Yes

Are the datasets clearly presented in a useable and accessible format?

Yes

Are the protocols appropriate and is the work technically sound?

Yes

Reviewer Expertise:

Arthropod genomics

I confirm that I have read this submission and believe that I have an appropriate level of expertise to confirm that it is of an acceptable scientific standard.

# Reviewer report: Reviewer response for version 1

**DOI:** 10.21956/wellcomeopenres.25081.r104459

**Published:** 2024-11-01

**Authors:** Susana Campino

**Affiliations:** 1 London School of Hygiene and Tropical Medicine, London, UK

This manuscript describes the genome assembly of an individual *Nineta flava*, a Green Lacewing insect, collected from Tonbridge, Kent, UK.

 It would be helpful to clarify certain parts:

 1. Distinctive Features: The text states, “Adults of *N. flava* can be distinguished from other Green Lacewings in Britain and Ireland by the combined features of a green body….” Are these characteristics specific to *N. flava* individuals from Britain and Ireland, or are they general features that distinguish *N. flava* from Green Lacewings found throughout the Western Palaearctic region?

 2. Western Palaearctic Details: Consider adding a brief description or list of regions included in the Western Palaearctic.

 3. Abbreviation Clarification: Please define "IUCN."

 4. Sentence Ambiguity: The sentence “No Chrysopidae from Europe have at this moment been assessed for risk of extinction by the IUCN, but this common, well-researched species with a wide distribution has not been flagged as a conservation concern in the literature” is unclear. *N. flava* is not a current extinction risk in Europe, but it not a  conservation concern?  Or it a  conservation concern?

 5. Additionally, is there any concern for extinction risk outside of Europe?

 6. Available Genomes: Are there any other assembled genomes available for this species or other Green Lacewings? Providing this information would add valuable context.

 7.Rationale for Genome Creation: Could you elaborate on the motivation behind creating this genome? Is this the first genome assembly for this species in the UK? More details on the purpose of this dataset would strengthen the manuscript.

 8. Coverage Calculation: In the description, “The genome of an adult *Nineta flava* (Figure 1) was sequenced using Pacific Biosciences single-molecule HiFi long reads, generating a total of 31.47 Gb (gigabases) from 2.81 million reads, providing approximately 39-fold coverage. Primary assembly contigs were scaffolded with chromosome conformation Hi-C data, which produced 175.58 Gbp from 1,162.76 million reads, yielding an approximate coverage of 240-fold,” how was the coverage determined? Is there an existing genome size reference, or was this estimate based on preliminary assembly data?

 9. Methods: The methods section is well-structured, with clear references to protocols and relevant sources. Is the sequenced individual a male or female? Can you add this information please.

 10. Analysis: The analysis section provides helpful links to replicate the analysis. Are there other genomes available to add some comparison analysis?

 Overall it is a nice manuscript with need for some extra, small details. 

Are sufficient details of methods and materials provided to allow replication by others?

Yes

Is the rationale for creating the dataset(s) clearly described?

Partly

Are the datasets clearly presented in a useable and accessible format?

Yes

Are the protocols appropriate and is the work technically sound?

Yes

Reviewer Expertise:

Genomics of pathogens and vectors.

I confirm that I have read this submission and believe that I have an appropriate level of expertise to confirm that it is of an acceptable scientific standard.

# Reviewer report: Reviewer response for version 1

**DOI:** 10.21956/wellcomeopenres.25081.r97484

**Published:** 2024-09-25

**Authors:** Yash Gupta

**Affiliations:** 1 Naresuan University, Phitsanulok, Thailand

I have thoroughly reviewed your manuscript on the genome sequencing of *Nineta flava* and would like to offer some suggestions for enhancing the clarity and grammatical precision of the work. 

 Manuscript is well-structured and makes a meaningful contribution to the field of entomology and genomics.

**Weaknesses and Recommendations:**

- Depth of Background Information: While the background provides a good overview of *Nineta flava*, it would benefit from a more detailed discussion on the ecological significance and previous genomic studies (if any) within the Chrysopidae family.
- Comparison with Related Species: Comparing the assembly metrics (e.g., genome size, N50, BUSCO scores) with those of closely related species could provide context on the assembly quality and any species-specific genomic features.
- Haplotype Information: The manuscript mentions that the assembly is not fully phased, Clarifying the extent of haplotype representation and its potential impact on downstream analyses would be beneficial.
- The manuscript would benefit from a short discussion on how the *N. flava *genome can be utilized in future research, such as in studies of evolutionary biology, ecology, pest management, or conservation genetics.
- Quality Value (QV): Explain the significance of the reported Quality Value (QV) of 57.6 in lay terms for broader accessibility.
- Gap Analysis: While the number of gaps is reported, discussing the nature or potential impact of these gaps on gene annotation or other analyses would be informative.

**Method Weaknesses and Recommendations:**

- Provide more context about the collection environment, such as habitat description, weather conditions, and any relevant ecological information. This can enhance understanding of the specimen's origin and potential environmental DNA influences.
- Include details on the time between collection and preservation, and any steps taken to minimize DNA degradation (e.g., immediate freezing).
- Specify the primers used for COI amplification, PCR conditions, and sequencing platform. This information is crucial for reproducibility and verification by other researchers.
- Present the results of the barcode comparison, including sequence similarity percentages and accession numbers of matching entries in the BOLD database.
- Explain why head and thorax tissues were chosen for DNA extraction. If this choice was to reduce contamination or improve DNA quality, stating so would provide valuable context.
- **Create a flow chart for your method section  to visually summarize the workflow of your study. It can enhance readers' understanding by providing a clear, step-by-step representation of the processes involved.**

Are sufficient details of methods and materials provided to allow replication by others?

Partly

Is the rationale for creating the dataset(s) clearly described?

Yes

Are the datasets clearly presented in a useable and accessible format?

Yes

Are the protocols appropriate and is the work technically sound?

Partly

Reviewer Expertise:

Bioinformatics

I confirm that I have read this submission and believe that I have an appropriate level of expertise to confirm that it is of an acceptable scientific standard, however I have significant reservations, as outlined above.
